# A New Extension Bracket

**Published:** 1867-01

**Authors:** 


### A NEW EXTENSION BRACKET.

We recently put in use one of Snow & Lewis' new "Sliding Extension Brackets," and like it very much. Its chief advantages are that it can be shortened without the projections made by the angles of the ordinary hinge or joint bracket; this one can be shortened and still present the straight shaft or arm: and again, it is more firm than the hinge bracket. It consists of pieces of iron pipe, neatly fitted and sliding into each other. A neat table accompanies it, to which is attached a wooden cup for holding instruments, which is a thing of great convenience. They are for sale at the Dental Depots.

